# Prevalence, etiology and clinical characteristics of gingival recession in a sample of adult Egyptian dental patients: a cross sectional study

**DOI:** 10.1186/s12903-025-06020-3

**Published:** 2025-05-07

**Authors:** Susan Sarhan, Enji Ahmed, Radwa R. Hussein, Asmaa Abou-Bakr

**Affiliations:** 1 Oral Medicine and Periodontology, Faculty of Dentistry, Ain Shams University, Cairo, Egypt; 2 Oral Medicine and Periodontology, Faculty of Dentistry, Cairo University, Giza, Egypt; 3 Oral Medicine and Periodontology, Faculty of Dentistry, Galala University, Suez, Egypt

**Keywords:** Gingival recession, Prevalence, Periodontitis, Risk factors, Egypt

## Abstract

**Background:**

Gingival recession (GR) is a common oral health condition characterized by the exposure of the tooth's root which affects diverse populations worldwide. Thus, this study aimed to analyze data from adult dental patients at the outpatient clinic of the Faculty of Dentistry, Ain Shams University, to assess the prevalence of GR among Egyptian adults and to identify associated risk factors and clinical characteristics.

**Materials and methods:**

This study was a cross-sectional study that included 3773 individuals to detect the prevalence, risk factors and clinical characteristics of GR. All participants were examined for presence of GR, and their demographic data was collected by electronic form, while only participants who fulfilled the inclusion criteria were then subjected to a full professional periodontal examination. GR was categorized following the 2018 World Workshop Cairo classification system (RT1, RT2, RT3). The statistical tests used were Kruskal–Wallis and Dunn's post hoc with Bonferroni correction, as well as Fisher's exact test followed by pairwise comparisons using multiple z-tests with Bonferroni correction.

**Results:**

Out of 3773 participants only 901 subjects had GR with a prevalence of 23.88%. The prevalence of localized recession (63.93%) was higher than generalized recession (36.07%) with RT1 (43.8%) being the most prevalent followed by RT2 (37.29%), and RT3 (18.87%). The most affected teeth were lower anterior teeth (46.53%) followed by upper left premolars (13.02%), then upper right premolars (11.11%), and upper anterior teeth (10.76%). The prevalence of GR was higher in males (59.6%) more than in females (40.4%), and in smokers (61.49%) more than non-smokers (38.51%). The most common medical conditions were diabetes mellitus and hypertension. Higher severity of the GR was associated with males, middle aged and old age, periodontal diseases, higher plaque and bleeding scores, medical conditions, smoking, and uneducated patients.

**Conclusions:**

The prevalence of GR in Egyptians is 23.88% with the most prevalent class of was RT1. Higher severity of the GR was associated with periodontal diseases, higher plaque and bleeding scores, presence of underlying medical conditions and smoking suggesting that regular dental assessments as well as dentists' awareness of the prevention, and treatment of GR has to be increased.

**Supplementary Information:**

The online version contains supplementary material available at 10.1186/s12903-025-06020-3.

## Introduction

Gingival recession (GR) is the exposure of the root surface caused by apical migration of the gingival margin following clinical attachment loss. Although it is not associated with an increase in tooth loss, many patients are accompanied by esthetic and functional problems, as well as the presence of dentin hypersensitivity and carious/non-carious cervical lesions on the bare root surface [1, 2]. Furthermore, these associated oral consequences with GR could have a negative impact on the oral health-related quality of life of an individual, as it appears to impact physical and psychological disabilities, functional limitation, and cause pain [3].

GR has become a public health concern, but oral epidemiology has not given it enough attention. Meanwhile, GR is not recorded by the Community Periodontal Index of Treatment Needs (CPITN), which has been utilized in a number of various studies examining the population's periodontal or oral health status [4]. Since then, the majority of periodontology research and clinical work has focused on newer surgical techniques and biomaterials for exposed root surface coverage. This resulted in benefits that were primarily patient-related but did not contribute to the public health aspects of this condition [5, 6].

The prevalence and distribution of GRs have been previously studied in the literature with diverse results, according to a recent systematic review by Yadav et al. in 2023 [4]. GR affects more than three-fourths of the global population. Although the final evidence received a very low overall grade, which emphasizes the need for more studies on GR prevalence. Therefore, a better knowledge of the prevalence of GR, especially in representative population samples from different geographic areas, is anticipated to provide the information for public health and oral healthcare professionals needed to set up effective preventive and treatment plans; thus, it will help to fill this research gap [4].

GR is commonly observed in daily practice at dental clinics [7]. The frequency, extent, and severity of gingival recession vary significantly among different study populations [8]. Among Egyptians, gingival recession is a very common oral condition, with a prevalence varying between 32.8% and 69.4% [9–11].

GR is attributed to various etiological factors. These can be broadly classified into four main categories: anatomical, iatrogenic, pathological, and traumatic [12]. Anatomical factors include excessive frenum or muscle attachment, tooth malposition, bone deficiencies, absence of attached gingiva, and the presence of a specific gingival biotype [13]. Iatrogenic factors contributing to GR can include poorly designed partial dentures, as well as complications arising from dental treatments such as faulty orthodontic appliances [14]. Inflammatory periodontal diseases and bacterial plaque accumulation are considered the main pathological etiological factors, in addition to mechanical trauma from aggressive teeth brushing [15].

Moreover, smoking is considered an important environmental risk factor in GR [16]. Furthermore, a significant loss of periodontal tissue is linked to many systemic diseases like diabetes mellitus, hypertension, chronic renal diseases, and hyperparathyroidism [17, 18]. In addition to the general health status of the individual, the extent and severity of GR are further exacerbated by the immunocompromised health status, as those on immunosuppressive drugs such as methotrexate and steroids, or poor nutrition, particularly deficiencies in vitamins A, C, and zinc, which impair immune response and delay periodontal repair [19, 20].

Interactions between these factors may cause GR defects. In most cases, we were able to identify multiple etiological factors. As a result, having one of these factors may increase the risk of getting GRs [21]. The most common risk factors reported in the literature were age, gender, ethnicity, teeth brushing, tooth type, arch, plaque score, bleeding score, periodontitis stage, and educational level [21–23].

Even though gingival recession is a very common oral health problem in Egypt, there is insufficient nationally representative data on the prevalence of gingival recession and its related risk factors. According to the literature this is the first epidemiological study detecting the prevalence of GR in a sample of adult Egyptian dental patients using the RT classification system proposed by the most recent World classification workshop, Cairo et al. [24], as well as determining the different risk factors and clinical characterization of GR to establish a framework for future studies to build upon, enhancing the overall understanding of GR’s etiology and related risk factors to ensure the consistency and reliability in diagnosis, allowing comparison with other studies besides contributing a baseline essential information for designing preventative strategies and treatment guidelines tailored to this population.

Null Hypothesis (H₀): There is no significant association between gingival recession and factors such as age, gender, smoking, oral hygiene habits, and periodontal status in the Egyptian adult population. Alternative Hypothesis (H₁): Gingival recession is significantly associated with age, gender, smoking, oral hygiene habits, and periodontal status in the Egyptian adult population.

## Methods

### Study design

This was an observational analytical cross-sectional study that addressed the prevalence, risk factors, and clinical characteristics of gingival recession in a group of adult Egyptian dental patients. The outpatient clinic at the Faculty of Dentistry, Ain Shams University, was used to select participants. The study was carried out between November 2022 and May 2024.

### Ethical approval

This study followed the World Medical Association's Declaration of Helsinki. The Research Ethics Committee, Faculty of Dentistry, Ain Shams University in Egypt, granted approval under the number FDASU-REC IR092213. Every patient received detailed verbal and written information about the study protocol. The understanding and agreement to participate in the study were confirmed through a written consent for all participants. Individual patient data and results were kept confidential by a filing system that was password-protected to prevent them from being breached. The analyzed data did not include patient names.

The primary outcome of the current study was the prevalence of GR based on the Cairo classification system (RT1, RT2, RT3). The secondary outcomes included the assessment of associated risk factors such as oral hygiene habits, systemic conditions, smoking status, and demographic variables, as well as the clinical periodontal characteristics.

The inclusion criteria were both genders aged 20 and up, participants with any underlying systemic diseases, and those who were medically free.

The exclusion criteria were those who refused to participate, vulnerable groups such as pregnant females, mentally and physically handicapped individuals, and subjects with fewer than 20 remaining teeth, 3rd molars, and remaining roots.

### Participants recruitment

**Fig. 1:**
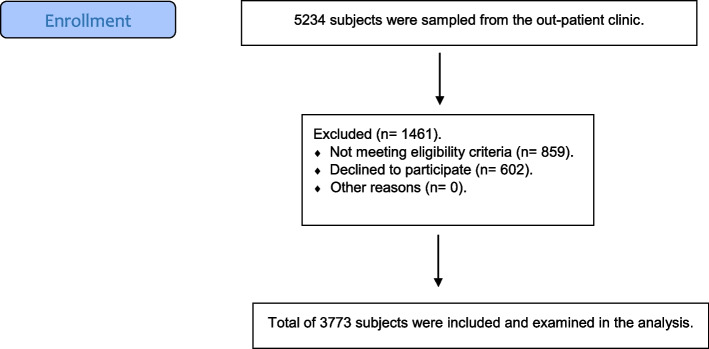
Flow diagram for study participants

To reach the targeted sample size, the adult Egyptian individuals who attended the outpatient clinic at the Faculty of Dentistry, Ain Shams University, were recruited consecutively. Then only those who fulfilled the eligibility criteria and agreed to participate in the current investigation were all examined by a well-trained and calibrated internal dental resident for the presence or absence of GR. The flow chart of the participant’s recruitment in the study is summarized in Fig. 1.

Afterwards, all participants enrolled in the study; they were asked to respond to a well-structured electronic form regarding their demographic data, medical history, social history, dental history, and personal habits [23]. The form was divided into three sections.

### Data collection form

The first section (demographic data) regarding the age (categorized in three groups: from 20 to 40 years (young adults), from 40 to 60 years (middle-aged adults), and above 60 (old adults)), gender, and educational level (as educated and non-educated) that was used as an indicator of socioeconomic status, cigarette smoking (categorized in three levels: heavy smoker (≥ 10 cigarettes/day), light smoker (< 10 cigarettes/day), and non-smoker) [12], and tooth brushing. The second section (medical condition status), which listed all of the possible chronic diseases that the patient may have, as well as whether the patient was medically free. The third section included the history of previous orthodontic treatment and any personal habits with the parafunctional habits addressed in the current study, referring to any abnormal hyperactivity of the temporomandibular system, for instance, bruxism, lip and nail biting, as well as mouth breathing [25].

### Clinical examination

Participants with GR underwent a professional clinical evaluation by two different expert and well-trained periodontists with more than 15 years of experience [S.S. and R.R] who both underwent a calibrated session on ten patients before the initiation of the study. All individuals were examined according to the new classification system that has classified gingival recessions with reference to the inter-dental clinical attachment loss, distinguishing among the three different types proposed by Cairo et al. [24] and Cortellini & Bissada [2] with an identical Williams graduated periodontal probe (Hu-Friedy, Chicago, IL, USA), to avoid measurement bias.

The distribution of the GR was evaluated as follows: localized or single recession when the patient had a single or 2 adjacent teeth with GR, while generalized or multiple recessions occurred when the patient had more than 2 non-adjacent teeth or had recession in all quadrants.

For patients with multiple teeth having different types of recession, each tooth had been categorized according to Cairo et al. [24] classification into either RT1, RT2, or RT3 class, and then we counted the total number of teeth with GR for each patient.

Each tooth's mesial, distal, buccal, and palatal surfaces were probed with a Williams periodontal probe to assess dental plaque thickness and calculate the plaque index. The average of the data collected for each tooth was used to estimate the individual's plaque index. The plaque index was calculated using the following reference values from Silness and Löe [26]. Bleeding on probing (BOP) was measured according to Lang et al. [27] for the presence or absence of bleeding.

The transparency test was used to assess gingival thickness at the recession site. The probe was inserted into the midfacial gingival sulcus. It was determined independently whether the probe was visible through the gingival tissue after being inserted into the gingival sulcus.

A pull test was performed by lifting the lip outwards to determine the presence of pull syndrome and the impact of high frenum attachment (gingiva blanching indicates high frenum attachment). With this pulling, the gingival edge opens, and the interdental papilla shifts. To determine whether tooth brushing was improper or excessive, the facial surface of each tooth was checked for cervical abrasion [28].

## Sample size calculation

Three thousand seven hundred seventy-three subjects were participating in this study according to a power analysis that was calculated based on the results of a previous study by Abou El Fadl et al. in 2021 [29]. Sample size calculation was performed using G*Power version 3.1.9.4.

### Statistical analysis

Inter-rater reliability was evaluated according to variable type: numerical variables (e.g., CAL) were assessed using a two-way mixed-effects intra-class correlation coefficient (ICC); ordinal variables (e.g., recession severity and plaque score) with the weighted kappa coefficient; and categorical variables (e.g., bleeding on probing) using Cohen’s kappa coefficient. Categorical and ordinal data were presented as frequencies and percentages. Associations between various risk factors and recession severity were analyzed using Kruskal–Wallis's and Dunn’s post hoc tests with Bonferroni correction. Associations between various risk factors and recession distribution were analyzed using Fisher's exact test followed by pairwise comparisons using multiple z-tests with Bonferroni correction. Effect sizes were calculated to assess the degree of associations between gingival recession (GR) and other variables in addition to statistical significance testing. A quantitative indicator of the degree or extent of a correlation, difference, or association between study variables is called effect size. Effect sizes show the magnitude or significance of an effect, in contrast to *p*-values, which merely show whether an impact occurs. For the Kruskal–Wallis test, the percentage of variance in GR severity that each component accounted for was measured using eta-squared (η^2^[H]) effect size [30]. Cohen's ω [31] was used to determine the degree of correlation between categorical variables for Fisher's exact test. By revealing effect sizes, one can gain a better understanding of the findings'practical significance and gain insight into the elements that significantly affect the severity of GR. Stepwise binary logistic regression models explored the relationship between recession severity classes and different risk factors. Model selection was based on the Akaike Information Criterion (AIC), choosing the model with the lowest AIC value. Leave-one-out cross-validation (LOOCV) was conducted before analysis to ensure the generalizability of the results. Linearity in the log odds was assessed using binned residual plots, which confirmed no significant deviations from linearity. Multicollinearity was evaluated with the Variance Inflation Factor (VIF), ensuring all predictors had VIF values below 5. Statistical analyses were performed using R software version 4.4.2 for Windows.^1^

## Results

### Demographic characteristics and clinical variables of the participants

The study group contained 537 (59.60%) males and 364 (40.40%) females. The age distribution was as follows: 136 (15.09%) were young, 312 (34.63%) were middle-aged, and 453 (50.28%) were old. Regarding their educational levels, 425 (47.17%) participants were uneducated, and 476 (52.83%) were educated. In terms of smoking status, 347 (38.51%) were non-smokers and 554 (61.49%) were smokers. Among the smokers, 216 (38.99%) were light smokers and 338 (61.01%) were heavy smokers, as in Table 1.

**Table 1:** Demographic data of participants with gingival recession

*Parameter (n* = *901)*		*n (%)*
Gender	Male	537 (59.60%)
	Female	364 (40.40%)
Age	Young adults	136 (15.09%)
	Middle-aged adults	312 (34.63%)
	Old adults	453 (50.28%)
Educational level	Not educated	425 (47.17%)
	Educated	476 (52.83%)
Smoking	No	347 (38.51%)
	Yes	554 (61.49%)
Number of cigarettes per day (*n* = 554)	Light smoker	216 (38.99%)
	Heavy smoker	338 (61.01%)

### General health status

Three hundred thirty-six (37.29%) cases were medically free, while 565 (62.71%) were medically compromised. The distribution of the different underlying medical conditions is as follows: Diabetes mellitus was present in 316 (35.07%) participants. Hypertension was observed in 265 (29.41%) participants. 12 (1.33%) participants reported thyroid problems. Cardiovascular disease (CVD) affected 45 (4.99%) participants. Chronic kidney disease (CKD) was present in 7 (0.78%) participants, as presented in Table 2.

**Table 2:** Different medical conditions

*Parameter (n* = *901)*		*n (%)*
Medical condition	Medically free	336 (37.29%)
	Medically compromised	565 (62.71%)
Diabetes mellitus	No	585 (64.93%)
	Yes	316 (35.07%)
Hypertension	No	636 (70.59%)
	Yes	265 (29.41%)
Thyroid problem	No	889 (98.67%)
	Yes	12 (1.33%)
CVD	No	856 (95.01%)
	Yes	45 (4.99%)
CKD	No	894 (99.22%)
	Yes	7 (0.78%)

### Prevalence of gingival recessions (GR)

**Fig. 2:**
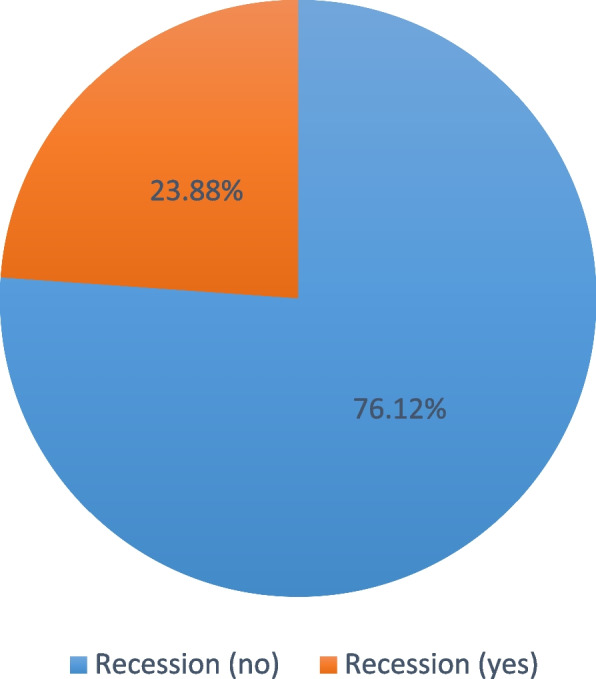
Pie chart showing recession prevalence

Among the 3773 examined participants, only 901 (23.88%) were diagnosed with gingival recession as presented in Fig. 2. Regarding recession classification, 395 (43.8%) participants were classified as RT 1, 336 (37.29%) as RT 2, and 170 (18.87%) as RT 3. The total number of teeth with GR in the studied participants was 3732. Teeth with RT1 GR were 1635, teeth with RT2 GR were 1391, and teeth with RT3 GR were 706.

### Clinical variables of the included participants with GR

Plaque scores among participants were as follows: 151 (16.76%) had a score of 0, 222 (24.64%) had a score of 1, 237 (26.30%) had a score of 2, and 291 (32.30%) had a score of 3. 395 (43.84%) had periodontal disease. Bleeding on probing was observed in 606 (67.26%) participants. The gingival biotype was classified as thin in 650 (72.14%) participants and thick in 251 (27.86%). 268 (29.74%) participants reported brushing their teeth as shown in Table 3.

**Table 3:** Clinical variables of the included participants

*Parameter (n* = *901)*		*n (%)*
***Plaque score***	***0***	151 (16.76%)
	***1***	222 (24.64%)
	***2***	237 (26.30%)
	***3***	291 (32.30%)
***Periodontal disease***	***No***	506 (56.16%)
	***Yes***	395 (43.84%)
***Bleeding on probing***	***No***	295 (32.74%)
	***Yes***	606 (67.26%)
***Gingival biotype***	***Thin***	650 (72.14%)
	***Thick***	251 (27.86%)
***Teeth brushing***	***No***	633 (70.26%)
	***Yes***	268 (29.74%)

### Recession distribution and causes

576 (63.93%) diagnosed cases had localized recession, and 325 (36.07%) had generalized recession. Among those with localized recession (n = 576), distribution by tooth was as follows: lower anterior: 268 (46.53%), upper anterior: 62 (10.76%), lower right premolars: 44 (7.64%), lower left premolars: 46 (7.99%), upper right premolars: 64 (11.11%), upper left premolars: 75 (13.02%), lower right molars: 3 (0.52%), lower left molars: 4 (0.69%), upper right molars: 5 (0.87%), and upper left molars: 5 (0.87%). The distribution of localized recession by quadrant (n = 576) was as follows: anterior: 330 (57.29%), right: 116 (20.14%), and left: 130 (22.57%). Distribution by arch (n = 576) was lower: 365 (63.37%) and upper: 211 (36.63%) all presented in Table 4.

**Table 4:** Recession distribution, causes, and associated problems

*Parameter*		*n (%)*
***Type of recession (n* = *901)***	***Localized***	576 (63.93%)
	***Generalized***	325 (36.07%)
***Distribution of localized recession by tooth (n* = *576)***	***Lower anterior***	268 (46.53%)
	***Upper anterior***	62 (10.76%)
	***Lower right premolars***	44 (7.64%)
	***Lower left premolars***	46 (7.99%)
	***Upper right premolars***	64 (11.11%)
	***Upper left premolars***	75 (13.02%)
	***Lower right molars***	3 (0.52%)
	***Lower left molars***	4 (0.69%)
	***Upper right molars***	5 (0.87%)
	***Upper left molars***	5 (0.87%)
***Distribution of localized recession by quadrant (n* = *576)***	***Anterior***	330 (57.29%)
	***Right***	116 (20.14%)
	***Left***	130 (22.57%)
***Distribution of localized recession by arch (n* = *576)***	***Lower***	365 (63.37%)
	***Upper***	211 (36.63%)
***Causes***	***Faulty restorations***	9 (1.00%)
	***High frenal attachment***	67 (7.44%)
	***Occlusal trauma***	158 (17.54%)
	***Orthodontic treatment***	51 (5.66%)
	***Parafunctional habits***	105 (11.65%)
	***Periodontal disease***	395 (43.84%)
	***Trauma from brushing***	118 (13.10%)
	***Others***	30 (3.33%)

The contributing factors (n = 576) were as follows: faulty restorations: 9 (1.00%), high frenal attachment: 67 (7.44%), occlusal trauma: 158 (17.54%), orthodontic treatment: 51 (5.66%), parafunctional habits: 105 (11.65%), periodontal disease: 395 (43.84%), trauma from brushing: 118 (13.10%), and others: 30 (3.33%) as in Table 4.

### Associations with different RT classes

**Fig. 3:**
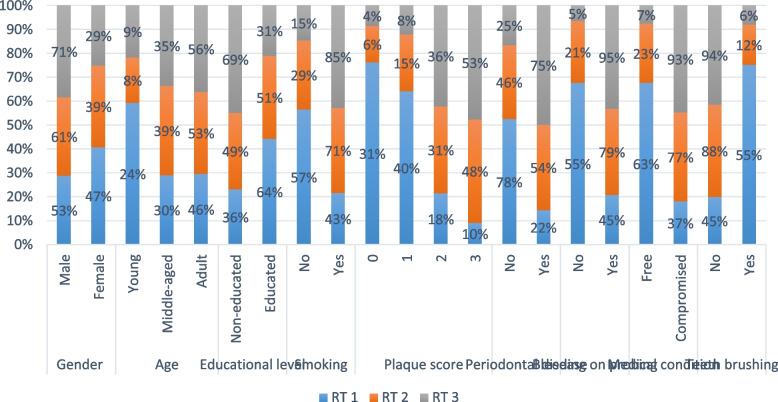
Bar chart showing associations with recession severity

As shown in Table 5 and Fig. 3, all associations with recession severity were statistically significant (*p* < 0.001). Results showed that higher severity was observed in males than in females. Middle-aged and older cases were more likely to have higher severity than younger cases. Additionally, uneducated cases were found to have a higher severity of recession than educated cases. The same was true for smokers and medically compromised cases. Higher severity of the recession was also associated with higher plaque scores, periodontal disease, bleeding on probing, and not brushing teeth.

**Table 5:** Associations with different types of recession

Parameter		Recession classification [n (%)]			*p*-value	Effect size	
		RT 1	RT 2	RT 3		eta2[H] (95% CI)	Magnitude
**Gender**	**Male (A)**	211 (53.42%)	205 (61.01%)	121 (71.18%)	< 0.001*	**0.016 (0.003:0.040)**	**Small**
	**Female (B)**	184 (46.58%)	131 (38.99%)	49 (28.82%)			
**Age**	**Young (B)**	95 (24.05%)	26 (7.74%)	15 (8.82%)	< 0.001*	**0.038 (0.020:0.070)**	**Small**
	**Middle-aged (A)**	120 (30.38%)	132 (39.29%)	60 (35.29%)			
	**Adult (A)**	180 (45.57%)	178 (52.98%)	95 (55.88%)			
**Educational level**	**Non-educated (A)**	141 (35.70%)	166 (49.40%)	118 (69.41%)	< 0.001*	**0.057 (0.030:0.090)**	**Small**
	**Educated (B)**	254 (64.30%)	170 (50.60%)	52 (30.59%)			
**Smoking**	**No (B)**	225 (56.96%)	98 (29.17%)	25 (14.71%)	< 0.001*	**0.120 (0.080:0.160)**	**Moderate**
	**Yes (A)**	170 (43.04%)	238 (70.83%)	145 (85.29%)			
**Plaque score**	**0 (C)**	124 (31.39%)	21 (6.25%)	6 (3.53%)	< 0.001*	**0.296 (0.250:0.350)**	**Large**
	**1 (C)**	159 (40.25%)	50 (14.88%)	13 (7.65%)			
	**2 (B)**	72 (18.23%)	104 (30.95%)	61 (35.88%)			
	**3 (A)**	40 (10.13%)	161 (47.92%)	90 (52.94%)			
**Periodontal disease**	**No (B)**	309 (78.23%)	155 (46.13%)	42 (24.71%)	< 0.001*	**0.177 (0.130:0.230)**	**Large**
	**Yes (A)**	86 (21.77%)	181 (53.87%)	128 (75.29%)			
**Bleeding on probing**	**No (B)**	216 (54.68%)	70 (20.83%)	9 (5.29%)	< 0.001*	**0.181 (0.140:0.230)**	**Large**
	**Yes (A)**	179 (45.32%)	266 (79.17%)	161 (94.71%)			
**Medical condition**	**Free (B)**	247 (62.53%)	77 (22.92%)	12 (7.06%)	< 0.001*	**0.221 (0.180:0.270)**	**Large**
	**Compromised (A)**	148 (37.47%)	259 (77.08%)	158 (92.94%)			
**Teeth brushing**	**No (A)**	178 (45.06%)	295 (87.80%)	160 (94.12%)	< 0.001*	**0.220 (0.170:0.270)**	**Large**
	**Yes (B)**	217 (54.94%)	41 (12.20%)	10 (5.88%)			

Categories with different superscript letters within the same parameter are significantly different* Significant

All associations with recession distribution were statistically significant (*p* < 0.001). Results showed that generalized recession was significantly associated with lower educational level, smoking, higher plaque scores, periodontal disease, bleeding on probing, compromised medical condition, and the lack of teeth brushing as presented in Table 6.

**Table 6:** Associations with different types recession distribution

Parameter		Recession distribution [n (%)]		*p*-value	Effect size	
		Localized	Generalized		Cohen’s ω (95% CI)	Magnitude
**Educational level**	**Non-educated**	230 (39.93%)^A^	195 (60.00%)^B^	< 0.001*	**0.193 (0.128:0.258)**	**Small**
	**Educated**	346 (60.07%)^A^	130 (40.00%)^B^			
**Smoking**	**No**	254 (44.10%)^A^	94 (28.92%)^B^	< 0.001*	**0.150 (0.084:0.215)**	**Small**
	**Yes**	322 (55.90%)^A^	231 (71.08%)^B^			
**Plaque score**	**0**	149 (25.87%)^A^	2 (0.62%)^B^	< 0.001*	**0.394 (0.325:0.456)**	**Moderate**
	**1**	166 (28.82%)^A^	56 (17.23%)^B^			
	**2**	123 (21.35%)^A^	114 (35.08%)^B^			
	**3**	138 (23.96%)^A^	153 (47.08%)^B^			
**Periodontal disease**	**No**	497 (86.28%)^A^	9 (2.77%)^B^	< 0.001*	**0.808 (0.743:0.874)**	**Large**
	**Yes**	79 (13.72%)^A^	316 (97.23%)^B^			
**Bleeding on probing**	**No**	250 (43.40%)^A^	45 (13.85%)^B^	< 0.001*	**0.302 (0.237:0.368)**	**Moderate**
	**Yes**	326 (56.60%)^A^	280 (86.15%)^B^			
**Medical condition**	**Free**	289 (50.17%)^A^	47 (14.46%)^B^	< 0.001*	**0.355 (0.289:0.420)**	**Moderate**
	**Compromised**	287 (49.83%)^A^	278 (85.54%)^B^			
**Teeth brushing**	**No**	321 (55.73%)^A^	312 (96.00%)^B^	< 0.001*	**0.423 (0.358:0.488)**	**Moderate**
	**Yes**	255 (44.27%)^A^	13 (4.00%)^B^			

Values with different superscripts within the same horizontal row are significantly different* Significant

### Binary logistic regression models for different RT classes

For RT1, the overall model was statistically significant (χ^2^(11) = 511.74, *p* < 0.001), and the predictors explained 58.1% (Nagelkerke R^2^). Results showed that females, smokers, medically compromised, and periodontally affected cases all had significantly lower odds of having RT1. Additionally, they showed that middle- and older-aged patients had significantly higher odds of having RT1 than younger patients. They also showed that an increase in plaque score was significantly associated with lower odds of RT1. Finally, they showed that teeth brushing was significantly associated with higher odds of RT1, as presented in Table 7**.**

**Table 7:** Binary logistic regression model for RT1

Term	Odds ratio	95% CI		Test statistic	*p*-value
		**Lower**	**Upper**		
**Sex (female)**	0.44	0.28	0.69	**− 3.54**	< 0.001*
**Age (middle)**	8.16	3.91	17.67	**5.47**	< 0.001*
**Age (old)**	70.53	26.68	200.93	**8.28**	< 0.001*
**Education (educated)**	1.47	0.94	2.29	**1.68**	**0.093**
**Smoker (yes)**	0.22	0.14	0.34	**− 6.54**	< 0.001*
**Plaque score**	0.18	0.12	0.27	**− 7.9**	< 0.001*
**BOP (yes)**	1.96	0.96	4.05	**1.83**	**0.067**
**Medically compromised (yes)**	0.25	0.15	0.39	**− 5.9**	< 0.001*
**Teeth brushing (yes)**	2.8	1.48	5.36	**3.15**	0.002*
**Gingival biotype (thick)**	0.65	0.42	1.00	**− 1.96**	**0.051**
**Periodontal disease (yes)**	0.55	0.36	0.84	**− 2.78**	0.005*

*CI* Confidence interval, * significant (*p* < 0.05)

While regarding RT2, the overall model was statistically significant χ^2^(9) = 178.25, *p* < 0.001, and the predictors explained 24.5% (Nagelkerke R^2^). Results showed that females, educated cases, and smokers had significantly higher odds of having RT2. Additionally, they showed that old patients had significantly lower odds of having RT2 than younger patients. The same was true for cases with bleeding on probing and those brushing their teeth. Finally, they showed that an increase in plaque score was significantly associated with higher odds of RT2, as presented in Table 8.

**Table 8:** Binary logistic regression model for RT2

*Term*	*Odds ratio*	*95% CI*		Test statistic	*p*-value
		***Lower***	***Upper***		
***Sex (female)***	1.64	1.14	2.37	**2.64**	0.008*
***Age (middle)***	0.57	0.3	1.08	**− 1.72**	**0.085**
***Age (old)***	0.26	0.12	0.55	**− 3.45**	< 0.001*
***Education (educated)***	1.72	1.20	2.49	**2.93**	0.003*
***Smoker (yes)***	1.54	1.05	2.25	**2.21**	0.027*
***Plaque score***	3.77	2.65	5.48	**7.15**	< 0.001*
***BOP (yes)***	0.22	0.11	0.41	**− 4.62**	< 0.001*
***Medically compromised (yes)***	1.36	0.91	2.03	**1.52**	**0.129**
***Teeth brushing (yes)***	0.36	0.20	0.64	**− 3.40**	< 0.001*

*CI* Confidence interval, * significant (*p* < 0.05)

The overall mode for RT3 was statistically significant χ^2^(8) = 261.02, *p* < 0.001, and the predictors explained 40.5% (Nagelkerke R^2^). Results showed that smokers, cases with bleeding on probing, medically compromised cases, and those with periodontal disease all had significantly higher odds of having RT3. Additionally, they showed that middle-aged and old patients had significantly lower odds of having RT3 than younger patients. The same was true for educated cases as presented in Table 9.

**Table 9:** Binary logistic regression model for RT3

Term	Odds ratio	95% CI		Test statistic	*p*-value
		**Lower**	**Upper**		
**Age (middle)**	0.06	0.02	0.18	**− 4.87**	< 0.001*
**Age (old)**	0.01	0	0.04	**− 6.58**	< 0.001*
**Education (educated)**	0.18	0.1	0.32	**− 5.73**	< 0.001*
**Smoker (yes)**	3.25	2.01	5.42	**4.67**	< 0.001*
**BOP (yes)**	13.79	5.59	39.57	**5.3**	< 0.001*
**Medically compromised (yes)**	8.09	3.9	18.33	**5.33**	< 0.001*
**Gingival biotype (thick)**	1.45	0.94	2.21	**1.7**	**0.088**
**Periodontal disease (yes)**	2.63	1.68	4.19	**4.15**	< 0.001*

*CI* Confidence interval, * significant (*p* < 0.05)

For all measured outcomes, there was an excellent agreement (> 0.9) between both examiners that was statistically significant (*p* < 0.001) with the inter-examiner reliability presented in Table 10.

**Table 10:** Inter-examiner reliability

Parameter	Reliability coefficient (95% CI)	*p*-value
Buccal CAL	**0.988 (0.982:0.992)**^**1**^	< 0.001*
Interproximal CAL	**0.999 (0.998:0.999)**^**1**^	< 0.001*
Plaque index	**1 (1:1)**^**2**^	< 0.001*
Recession severity	**1 (1:1)**^**2**^	< 0.001*
Bleeding on probing	**1 (1:1)**^**3**^	< 0.001*

1 ICC, 2 Weighted Kappa, 3 Cohen's Kappa, CI Confidence interval, * significant (*p* < 0.05)

Strong effect sizes accompanied the statistically significant associations found between GR severity and variables like smoking, periodontal disease, plaque scores, and bleeding on probing. Notably, the high eta-squared (η^2^[H]) values for these variables show that they represent a significant amount of the variance in GR severity. Similarly, a strong correlation between smoking status, periodontal disease, and GR severity is shown by high Cohen's ω values for categorical comparisons. These results highlight how crucial it is to focus on these factors in both therapeutic and preventative strategies.

## Discussion

GR is a common and undesirable condition affecting individuals worldwide of all ages [22]. Few research studies that are currently accessible globally have focused on the existence of these circumstances in the aesthetic zone or have distinguished between the different types (RT1, RT2, and RT3) recommended by the 2018 classification system [13], which highlights the importance of interproximal attachment level, one of the key site-related prognostic factors, and offers a straightforward way to classify gingival recession in order to effectively predict treatment outcomes [24]. Therefore, the current research aimed to detect the prevalence of GR and associated risk factors in a sample of adult Egyptian dental patients using the RT classification system.

The findings of the present study indicated a significant association between GR and factors such as increasing age, smoking, and oral hygiene habits, supporting the alternative hypothesis (H₁) and rejecting the null hypothesis (H₀), which assumed no significant relationship.

Out of the 3773 subjects examined in the present study, 901 subjects had GR with a prevalence of 23.88%, and this was in agreement with previous studies that reported a prevalence of GR (< 50%) [9, 10, 28, 32–34]. Whereas a higher prevalence of (> 50%) gingival recession was seen in other studies [9, 16, 23, 35–37]***.*** This low frequency in Egypt could be explained by the preventive and awareness activities implemented by the public dental service in the previous several years.

In the current study, 537 (59.6%) of participants with GR were males, while 364 (40.4%) were females, with no significant difference between both genders. Which was in agreement with previous studies [9, 11, 23, 28, 35, 38]. On the contrary, other studies showed a female predominance [37, 39, 40]. The higher prevalence of GR in males can be attributed to a lower concern with oral hygiene and esthetics.

In the present study, frequency of gingival recession was found to increase with age. The young adults’ group (20–40 years) was 15.09%, followed by the middle adults’ group (40–60 years) at 34.63%, with the higher prevalence in older adults (above 60) at 50.28%. It was in accordance with previous studies, which found that the higher prevalence of GR in the older age group [21, 28, 37, 41–43]. While other investigators have found that in most of the studies, the frequency of recession was 100% for the older age group [32, 35]. A possible explanation for the association between gingival recession and age is that the agents responsible for gingival recession are exposed for longer periods of time; in addition to the cumulative effects of the lesion, intrinsic changes occur both locally and systemically within the body. However, a more widespread distribution evident in older participants may point to the combined impact of multiple factors, including previous periodontal disease accompanied by dental brushing trauma [28].

Regarding the educational level, educated participants with gingival recession were more prevalent (52.83%) than those who were not educated (47.17%), which was in line with earlier reports [21–23, 36, 40, 44–46], which suggested a positive association between high educational level and occurrence of gingival recession.

The significance of regular dental office visits and appropriate plaque control at home in maintaining oral health conditions may be more understood by educated individuals than by their less educated counterparts; thus, they are more likely to perform teeth brushing, and as assumed by previous studies, aggressive or inappropriate brushing techniques greatly increased the possibility of recession by inducing mechanical trauma [20, 28, 47], which could be an explanation for the present finding in our study.

Generally, it could be concluded that highly educated persons are more likely to experience RT1 recessions, while people with moderate and low levels of education are more likely to experience RT2 or RT3 recessions, and this was in line with a previous study [23]. This finding is consistent with the current study, as the percentage of educated people who experience RT1 recessions is 64.3%, while the percentage of people who experience RT2 or RT3 recessions is 50.6% and 30.59%, respectively.

Prevalence of GR was found to be higher in smokers (61.49%) than in non-smokers (38.51%), which was in line with previous studies [40, 41, 43, 48]. On the contrary, other studies didn’t identify smoking as a risk factor for the development of GR [21, 23, 37, 49].

Smokers have a higher prevalence of moderate and severe periodontitis than nonsmokers [50]. Additional evidence supports the detrimental and dose-dependent impact of tobacco product intake on periodontal health. Smokers had a higher prevalence of Treponema denticola, which may account for at least some of the increased incidence and severity of periodontal tissue loss [51, 52]. And this was confirmed in the current study, as heavy smokers were more prevalent (61%) than light smokers (39%).

Smoking affects the severity of GR by increasing the release of inflammatory mediators, reducing the immunological host response, and promoting the growth of pathogenic microflora that led to a periodontal tissue destruction [53]. Smoking can also have an impact on host immunological and inflammatory responses, including the suppression of alkaline phosphatase synthesis by nicotine, the immunosuppressive effects of macrophages on cell-mediated immune responses, and the inhibition of human periodontal ligament fibroblast migration, which led to further tissue destruction [54].

The usage of tobacco has a negative impact on one's dental and general health. Tobacco use is now one of the biggest global public health issues affecting the overall health-related quality of life. Therefore, this highlights the importance of the dentist’s role in smoking cessation [55].

Regarding general health status, 336 participants (37.29%) were medically free, while 565 (62.71%) were medically compromised. The most commonly reported medical conditions were diabetes mellitus (35.07%) and hypertension (29.41%).

Poorly controlled diabetes mellitus impairs wound healing, increases inflammation, and alters immune responses, accelerating periodontal tissue damage and exacerbating gingival recession [56]. Hypertension can also negatively affect periodontal health by compromising blood flow, impairing healing, and increasing systemic inflammation, thus promoting periodontal tissue destruction and gingival recession. In addition to causing gingival overgrowth, some antihypertensive drugs, such calcium channel blockers, may also indirectly raise the risk of gingival recession by changing the shape of the gums and causing plaque to build up [57, 58]. Managing these systemic conditions is crucial for the comprehensive care of patients with periodontitis.

The morbidity and mortality rates of non-communicable chronic diseases, like hypertension, diabetes, chronic renal diseases, and coronary heart disease, have increased globally over the past few decades due to the continuous changes in socioeconomic dynamics and lifestyle choices as well as the growing difficulties posed by an aging population. This increase raises serious public health issues and has a substantial effect on people's quality of life everywhere [59].

Regarding the GR classification, it was found that RT1 GR (43.8%) was the most prevalent, followed by RT2 (37.29%), and the least was RT3 (18.87%); this is in line with previous studies, which found that RT1 and RT2 classes represented the majority of their sample [10, 42, 43], while Romano et al. [22] found that RT1 and RT3 were the most common among the Italian population. Furthermore, other studies found that RT2 and RT3 GR classes were the most common among Spain and Morocco, respectively [21, 37].

It is reasonable to assume that these differences could also be explained by different age ranges, periodontal profiles, potential ethnic or genetic determinants, oral hygiene practices, varying educational and socioeconomic status [60], and exposure to risk factors, even though they could also be partially attributed to differences in populations, unlike geographical areas, or different methodological protocols [23].

People's vulnerability to periodontal disease is impacted by genetic variation due to its about 50% heritability [61]. Additionally, different nutritional habits and behavioral risk factors, such as smoking and poor oral hygiene, are significant modifying factors that affect the progression of periodontal disease [62]. Nutritional factors play a crucial role in maintaining the balance between oral microorganisms and the host’s immune response, which influences the onset and progression of periodontal disease [63]. The relation between nutrition and oral health is well established, and numerous studies published in recent years have emphasized the association between nutrition and periodontal disease [64, 65].

The state of periodontal and gingival health can also be directly impacted by a person's oral health habits, knowledge, and attitudes [59]. There is a direct and indirect relationship between health status, health behavior, and health literacy [66].

Moreover, sociodemographic and socioeconomic factors, including age, sex, and place of residence, as well as poverty and a lack of education, are the primary determinants of oral health in developing nations like Egypt. Apart from the aforementioned considerations, the lack of dental services in primary healthcare facilities results in restricted or nonexistent access to quality dental treatment [67]. As reported in a study by Moussa et al. in 2020 [62], only 34.3% of the Egyptian population sample reported brushing their teeth. Nonetheless, the majority of those who claimed to clean their teeth but actually didn't have poor oral health. They verified the link between poor oral hygiene practices and oral conditions such as periodontal disease [61].

Regarding the distribution of GR, 63.93% of cases had localized recession, and 36.07% had generalized recession. The most affected teeth were lower anterior teeth (46.53%), followed by upper left premolars (13%), then upper right premolars (11.11%), and upper anterior teeth (10.76%). These findings were in line with earlier research that suggested GR was more common in mandibular anterior teeth [21, 23, 32, 37, 38, 68, 69]. On the other hand, other surveys [20, 51, 52] revealed a greater occurrence in the upper premolar and molar region. GR may be found more in teeth that are prominently positioned as the alveolar bone is thin or absent with inadequate keratinized mucosa, possibly as a result of the decreased connective tissue availability, which in turn results in a localized inflammatory reaction [28]. Therefore, the significant frequency observed in this area of the mouth may be explained by the thin scalloped biotype that normally defines the lower incisor area [49].

Participants with plaque score 3 showed the highest prevalence of GR (32.30%), and it was in line with previous studies [23, 40, 45, 49]. The present data are consistent with earlier research findings [37, 40, 70] showing a significant correlation between high levels of bacterial plaque and GRs (both localized and generalized types). It was proposed that the destruction of connective tissue is caused by a localized inflammatory process due to the accumulation of dental plaque, and when epithelial cells proliferate within the connective tissue, the epithelial surface subsides, resulting in gingival recession [71].

Regarding bleeding on probing, positive bleeding on probing (67.26%) showed a higher prevalence than negative bleeding (32.74%); this comes in line with previous studies [16, 23, 45, 49, 72]***.*** Gingival inflammation, which is thought to be a reversible form of periodontal disease, is indicated by bleeding upon probing. If untreated, it can lead to the destruction of connective tissue and proliferation of the epithelium into the site of destruction, thus leading to gingival recession [73].

Considering gingival biotypes, a thin gingival biotype was evident in 650 (72.14%) of the included participants, which is in agreement with previous studies [23, 44, 74, 75]***,*** while it was against a previous study by Fragkioudakis et al. [49].

It was discovered that the gingival thickness and alveolar bone thickness were directly correlated [76]. Therefore, recession is more likely to occur in people with thin gingiva and narrow keratinized tissue than in people with thick gingiva and wide keratinized tissue [74, 77]. Cortellini and Bissada [2] reported that a thin gingival biotype has been identified as a major risk factor for the development of GR. However, a correlation between the biotype and GR could not be established in the current investigation. While some clinical investigations [78, 79] have demonstrated a strong correlation between the presence of GR and a thin biotype, other studies [80, 81] have not found this correlation.

These differences may be explained by the biotype discrimination technique. The approach suggested by Kan et al. [82] was used in this investigation to characterize gingival biotype. Selecting an alternative biotype-discriminating method might provide a more coherent relationship. Furthermore, recent data indicate the existence of three separate biotypes [2]; this fact was not considered in the current investigation.

When several recession causes were examined, periodontal disease had the highest prevalence, with 395 (43.84%) cases reported. These findings supported those of Hegab et al. [9], who found that among a sample of adult Egyptians, periodontal disease had the highest frequency as a cause of GR with 90.8%. Similarly, Romano et al. [23] found that RT2 and RT3 GRs were associated with periodontitis 55.91%. Gingival recessions are caused by interdental attachment loss, which mostly affects RT3 due to periodontitis [21]. This might be because the patients in the sample population, who were selected from public university hospitals and had little financial means for good oral hygiene practices, had a low socioeconomic position.

All associations with recession severity were statistically significant (*p* < 0.001) in the current study, with higher severity observed in males than in females. Middle-aged and older cases were more likely to have higher severity than younger cases. Additionally, uneducated cases were found to have a higher severity of recession than educated cases. The same was true for smokers and medically compromised cases. Higher severity of the recession was also associated with higher plaque scores, periodontal disease, bleeding on probing, and not brushing teeth. GR management and prevention strategies should be incorporated into routine clinical treatment. Therefore, oral health programs and campaigns that target the prevention of periodontal disease as well as raise awareness towards the importance of oral hygiene practice may lower the prevalence of GR.

This comes in line with previous studies that proved a significant association between gingival recession and bleeding on probing [16, 72]. Also, age is related to the existence of GRs, according to the majority of risk indicators in prior research [16, 21–23, 37, 44]. A recent systematic review supported this finding, finding that the prevalence of GRs increases with age and that men are more likely to be affected than women [4].

Also, there may be a higher chance of developing GRs in those with damaged periodontium [70]. Other risk variables, including gender, cigarette smoking, high education, and brushing traumatized teeth, were also identified in previous epidemiological studies [21–23, 36, 44]. As per Dilsiz et al. [83] and Pires et al. [84], tobacco smoking is regarded as a risk factor linked to GR and one of the primary causes of destructive forms of periodontal disease.

Although the existence of associations is shown by statistical significance, the reported effect sizes show how strong those associations are. Factors like smoking, plaque scores, and periodontal disease have been shown to have significant impact sizes, indicating that they are important in the onset and severity of GR. Therefore, in clinical practice, GR incidence and severity could be considerably decreased by addressing these risk factors through focused therapies. Moreover, the identification of high-impact variables strengthens the necessity of customized public health approaches meant to reduce predictable causes of GR. This can assist the dental professionals in prioritizing therapies based on the most impactful elements.

Among the study limitations were its cross-sectional design, which precluded the assessment of many factors'cumulative effects on GR over time; thus, more longitudinal studies are needed to establish a causal relationship between GR and the identified risk factors. Additionally, the study did not thoroughly examine socioeconomic position, psychological aspects, food habits, or genetic factors, all of which would have affected the results.

Future research should be directed to fully comprehend the interactions between genetic and microbiological elements that contribute to GR development. Examining a person's genetic predisposition may reveal particular genetic markers or inherited traits that make them more vulnerable to GR. Furthermore, investigating how the oral microbiota functions and interacts with the host's immune system could reveal information about how microbes affect periodontal health. That potentially leads to the development of more accurate diagnostic instruments and focused treatment plans.

## Conclusions

The prevalence of GR in Egyptians is 23.88%, with the most prevalent class being RT1. Higher severity of the GR was found to be associated with periodontal diseases, higher plaque and bleeding scores, presence of underlying medical conditions, and smoking, suggesting that regular dental assessments as well as dentists'awareness of the prevention, management, and treatment of GR have to be increased. The findings of the present study can be utilized to address the public health elements of GR and to quantify its burden as well as gain more knowledge about its associated risk factors among the Egyptian population to prevent GR and create long-term prevention plans.

## Supplementary Information

Supplementary Material 1.

## Data Availability

The data that support the findings of this study are available from [S.S], but restrictions apply to the availability of these data,which were used under license for the current study, and so are not publicly available. Data are however available from the authors upon reasonable request and with permission of [S.S].

## Footnotes

1 R Core Team (2024). R: A language and environment for statistical computing. R Foundation for Statistical Computing, Vienna, Austria. URL https://www.R-project.org/.
