# Supplementary material for: Prevalence, etiology and clinical characteristics of gingival recession in a sample of adult Egyptian dental patients: a cross sectional study
Source: BMC Oral Health. 2025 May 7;25:691. doi: 10.1186/s12903-025-06020-3 (PMC12060494; doi:10.1186/s12903-025-06020-3)
Supplement: Supplementary file 1 — Supplementary Material 1. [file 12903_2025_6020_MOESM1_ESM.pdf]

Gingival recession

Prevalence of esthetic gingival recession (anterior & premolar teeth)
The purpose of this survey is to gather and analyze data to detect the prevalence of gingival recession in adult Egyptian dental patient and to determine the different risk factors and clinical characterization of gingival recession. (kindly be informed that submitting your responses to this form is considered an approval of your participation in the survey) . this questionnaire received the approval from the ethical committee, Faculty of dentistry , Ain Shams University.

* Indicates required question

Demographic data & Habits

1. 1) Age *

Mark only one oval.

- < 40 (young)
- 40-60 (middle age)
- \> 60 (old)

2. 2) Gender *

Mark only one oval.

- Male
- Female

3. 3) Educational level *

Mark only one oval.

- Low
- Middle
- High

4. 4) Cigarettes smoking *

Mark only one oval.

- Yes
- No

5. 5) Number of cigarettes *

Mark only one oval.

- Less than 10 /day
- More than 10 / day
- Non

6. 6) Tooth brushing *

Mark only one oval.

- Yes
- No

Medical condition

7. 7) Medical problem (You can chose more than one) *

Tick all that apply.

- [ ] Diabetes
- [ ] Hypertension
- [ ] Heart problem
- [ ] Liver disease
- [ ] Kidney disease
- [ ] Thyroid problem
- [ ] Autoimmune disease
- [ ] Other
- [ ] Medically free

Clinical examination

8. 8) Recession *

Mark only one oval.

- ( ) Localized
- ( ) Generalized

9. 9) If Localized , which tooth? *

Tick all that apply.

- [ ] Upper anterior
- [ ] Lower anterior
- [ ] Upper Rt. Premolar
- [ ] Upper Lt. Premolar
- [ ] Lower Rt. Premolar
- [ ] Lower Lt permolar
- [ ] Non

10. 10) Plaque index *

Mark only one oval.

- zero
- 1
- 2
- 3

11. 11) Bleeding on probing *

Mark only one oval.

- Yes
- No

12. 12) Gingival biotype *

Mark only one oval.

- Thin
- Thick

13. 13) RT gingival recession classification

Mark only one oval.

- RT 1
- RT 2
- RT 3

14. 14) Cause of recession (You can choose more than one)

Tick all that apply.

- [ ] Trauma from brushing
- [ ] Periodontal disease
- [ ] Parafunctional habits
- [ ] Highly frenum attachment
- [ ] Orthodontic treatment
- [ ] Cervical restorative margins
- [ ] Traumatic occlusal forces
- [ ] Others

This content is neither created nor endorsed by Google.

Google Forms
